# Enhanced production of napthoquinone metabolite (shikonin) from cell suspension culture of *Arnebia* sp. and its up-scaling through bioreactor

**DOI:** 10.1007/s13205-013-0149-x

**Published:** 2013-06-09

**Authors:** Komal Gupta, Shashank Garg, Joginder Singh, Manoj Kumar

**Affiliations:** 1 Department of Biotechnology, Lovely Professional University, Punjab, India; 2 NIT, Surathkal, Karnataka India; 3 School of Biotechnology and Bio sciences, Lovely Professional University, Punjab, India

**Keywords:** Secondary metabolites, Plant cell culture, Air-lift bioreactor, Shikonin

## Abstract

Cell culture in shake flask and air-lift bioreactor was carried out to exploit the potential of *Arnebia* sp. for napthoquinone metabolite production. Cell suspension cultures of *Arnebia* were established from friable callus in liquid MS medium supplemented with 6-benzylaminopurine (BAP) (10 μM) and indole-3-butyric acid (IBA) (5 μM). Growth kinetic studies were done by using settled cell volume and fresh/dry cell weight method. Suspension cultures were maintained by sub-culturing at 10 days interval. A two-stage culture system is employed using growth medium (GM) and modified M9 medium (production medium) for cell biomass and naphthoquinone pigment production, respectively. Results showed that cultivation of cells under dark conditions at room temperature (25 ± 2 °C) enhanced the cell biomass from 100 to 625 g l^−1^. The pigment production was also found to be increased in dark conditions at room temperature. Alkaline pH found to have positive effect on pigment yield. In case of M9 medium constituents, absence of Na_2_SO_4_ does not affect the pigment yield. The current approaches have the cumulative effect to meet an increased level of (25.5 μg/ml) metabolite production in air-lift bioreactor.

## Introduction

The Indian Himalayan region (IHR) is a rich reservoir of biodiversity in the world. Over 1,748 species of medicinal and aromatic plants reported from IHR are used in different systems of medicine (Kandari et al. 2008). There are two types of plants derived molecules: (1) primary metabolites, and (2) secondary metabolites. Primary metabolites such as nucleic acids, proteins, carbohydrates and fats are primary compounds because of their roles in basic cell metabolism, and are usually “high volume low value” chemicals. Secondary metabolites are biosynthetically derived from primary compounds and are more limited in distribution and produced in smaller quantities. They tend to be synthesized in specialized cell types and at distinct development stages and difficult to synchronize the extraction and purification of these metabolites. Secondary metabolites such as shikonin, anthocyanin, hyoscyamine, vincristine, etc., has no apparent physiological function, but have ecological roles, e.g., attractant and chemical defense. Human use secondary metabolites as medicines, flavorings, and recreational drugs. Secondary metabolites are “high value low volume” products (Katare et al. 2009; Verpoorte and Alfermann 2000; Babula et al. 2009).

*Arnebia* (family Boraginaceae), commonly known as “Ratanjot”, is an important and valuable plant species found at high altitude of Western Himalayan region (4,000–4,200 m amsl). It has been used as a natural dye for coloring silk and cosmetics, food additives and medicine. Its roots possess naphthoquinone pigments or shikonin derivatives having medicinal properties such as antimicrobial, anticancer, antipyretic and anti-inflammatory (Saker et al. 2012; Verpoorte et al. 2002; Manjkhola and Dhar 2002). These properties of plant are attributed to the presence of shikonin derivatives. Owing to these properties, genus *Arnebia* is over-exploited and therefore, placed under the category of critically endangered species. The plants are very difficult to cultivate through conventional agriculture (possess third level of difficulty in seed germination) (Malik et al. 2011).

In this regard, plant cell culture seems to be a promising technique for production of naphthoquinone pigments under in vitro conditions (Onrubia et al. 2012; Verpoorte et al. 2002; Sharma et al. 2008). Several attempts have been made in recent past to enhance the production level which are very much significant for phytotherapeutical applications (Hussain et al. 2012).

In consideration of the above facts, the present study was undertaken with an aim to produce naphthoquinone pigment through cell culture of *Arnebia* sp. and its scale-up from shake-flask to air-lift bioreactor. This will not only help in meeting the industrial demand of these metabolites, but also lead to the conservation of the useful species in their natural habitat.

## Materials and methods

### Establishment of in vitro callus culture

#### Culture media, plant growth regulators and glassware

Murashige and Skoog (MS) medium (Chattopadhyay et al. 2002) was used for induction of callus from different explants. Sucrose (3 %; w/v) was added as a carbohydrate source and agar (0.8 %; w/v) was incorporated as a gelling agent. Different plant growth regulators (PGRs), i.e., kinetin (Kn) and 2,4-dichlorophenoxyacetic acid (2,4-D), 6-benzylaminopurine (BAP) and indole-3-butyric acid (IBA), BAP and 2,4-D, 2,4-D and 3, 6-dichloro-2-methoxybenzoic acid (DICAMBA) were used in factorial combinations (Table 1). PGR-free media served as control. The pH of the media was adjusted to 5.75 prior to autoclaving.

**Table 1:** Combinations of PGRs used for studying the effect of salts on callus induction of *Arnebia*

PGR	2,4-D			
	0 μM	2 μM	5 μM	10 μM
Kn				
0 μM	0,0 (T1)	0,2 (T2)	0,5 (T3)	0,10 (T4)
2 μM	2,0 (T5)	2,2 (T6)	2,5 (T7)	2,10 (T8)
5 μM	5,0 (T9)	5,2 (T10)	5,5 (T11)	5,10 (T12)
10 μM	10,0 (T13)	10,2 (T14)	10,5 (T15)	10,10 (T16)

PGR	IBA			
	0 μM	2 μM	5 μM	10 μM
BAP				
0 μM	0,0 (T17)	0,2 (T18)	0,5 (T19)	0,10 (T20)
2 μM	2,0 (T21)	2,2 (T22)	2,5 (T23)	2,10 (T24)
5 μM	5,0 (T25)	5,2 (T26)	5,5 (T27)	5,10 (T28)
10 μM	10,0 (T29)	10,2 (T30)	10,5 (T31)	10,10 (T32)

PGR	2,4-D			
	0 μM	2 μM	5 μΜ	10 μM
BAP				
0 μM	0,0 (T33)	0,2 (T34)	0,5 (T35)	0,10 (T36)
2 μM	2,0 (T37)	2,2 (T38)	2,5 (T39)	2,10 (T40)
5 μM	5,0 (T41)	5,2 (T42)	5,5 (T43)	5,10 (T44)
10 μM	10,0 (T45)	10,2 (T46)	10,5 (T47)	10,10 (T48)

PGR	2,4-D			
	0 μM	2 μM	2.5 μM	5 μM
Dicamba				
0 μM	0.0 (T49)	0,2 (T50)	0,2.5 (T51)	0,5 (T52)
2 μM	2,0 (T53)	2,2 (T54)	2,2.5 (T55)	2,5 (T56)
2.5 μM	2.5,0 (T57)	2.5,2 (T58)	2.5,2.5 (T59)	2.5,5 (T60)
5 μΜ	5,0 (T61)	5,2 (T62)	5,2.5 (T63)	5,5 (T64)

#### Plant material and sterilization

Leaf explants excised from the young plants growing in polyhouse were washed in running tap water for 5–10 min. The explants (5–7 cm) were rinsed in distilled water having 1–2 drops of liquid detergent (Teepol or Tween-20) for 10 min followed by 5 min washing with distilled water. The leaves were then surface sterilized with bavistin (0.02 % w/v) and streptomycin (0.02 % w/v) for 10 min followed by washing with distilled water thrice. The explants were then disinfected by 70 % ethyl alcohol treatment for 30–40 s. The leaves were then treated with 0.01 % HgCl_2_ (having 1–2 drops of tween-20) for 5 min followed by five washings with sterile distilled water inside the laminar airflow.

#### Inoculation

The leaf explants were dissected into small pieces (1.0–1.5 cm) and were inoculated into petri plates placing adaxial side in contact with solid MS medium with and without growth regulators. Cultures were kept at 25 ± 2 °C under dark condition.

*Callus induction and proliferation* The percentage for callus induction can be calculated by the following formula:

$$\text{Callus}\;\;\text{induction}\;\;(\%)=\frac{\text{Number of calli}}{\text{Number of explants inoculated}}\times 100.$$

### Establishment of cell suspension culture

The suspension cultures were established using growth medium (GM) (MS medium supplemented with BAP (10 μM) and IBA (5 μM)) as earlier standardized at the host institute. Sucrose (3 % w/v) was added as carbohydrate source. The pH of media was adjusted to 5.75 prior to autoclaving. The cultures were kept on shaker set at 100 rpm, 25 ± 2 °C under dark conditions and maintained with regular sub-culturing at 10 days interval.

#### Growth kinetics

The following methods were employed for studying the growth kinetics:

- *Settled cell volume* (*SCV*) SCV was determined by allowing a cell suspension to sediment in the side arm of the flask. It is reported as the percentage of the total volume of suspension occupied by the cell mass.
- *Fresh and dry cell weight* To measure fresh weight cell biomass was harvested by sieving suspension cultures through 40 μm mesh and weighed on a balance immediately, to reduce variations caused by water evaporation. Dry weight was estimated by drying the fresh tissue at 60 °C in a convection oven, until it reached constant weight (usually 12–14 h). Fresh weight was reported as gram, fresh weight per liter (g, FW l^−1^), while dry weight was reported as gram, dry weight per liter (g, DW L^−1^).

### Pigment production

A two-stage culture system was adopted, i.e., cell growth in GM and pigment in modified M9 medium, i.e., production medium (PM) (Katare et al. 2009). Sucrose (5 %; w/v) was added as carbohydrate source. The pH of the media was adjusted to 6.00 prior to autoclaving.

### Physical factors versus cell growth and pigment production

To study the effect of different factors on cell growth and pigment production in cell suspension culture, the cells from growth medium were inoculated at 10 % inoculum density in GM and PM. The cultures were kept on shaker set at 100 rpm, temperature at 25 ± 2 °C and incubated under required illumination conditions.

#### Light conditioning

To study the effect of light, cell suspension cultures were incubated under continuous light (photosynthetic photon flux density (PPFD), 70 μmol m^−2^ s^−1^) or complete darkness. Differential exposures of illumination period on pigment production from cell suspension cultures were determined (Table 2).

**Table 2:** Effect of light exposure duration on shikonin production

Replicates	Shikonin (μg/ml)	
	Mean	SD
R1 = treatment under 2 days dark + 8 days light	11.1	1.249
R2 = treatment under 4 days dark + 6 days light	10.75	1.671
R3 = treatment under 6 days dark + 4 days light	24.95	2.144
R4 = treatment under 8 days dark + 2 days light	12.95	2.308
R5 = treatment under 10 days dark	11.90	1.61
R6 = treatment under 10 days light	6.00	0.655

#### Effect of pH

To investigate the effect of pH on cell growth and pigment production, cells were incubated in GM and PM. with varied pH (ranging from 3.75 to 9.75). The pH of the media was adjusted with 0.1 N HCl or 0.1 M KOH prior to autoclaving. The flasks were incubated in dark condition at 25 ± 2 °C on shaker set at 100 rpm. Three replicates were used for each treatment.

### M9 medium constituents and pigment production

To investigate the effect of individual component of M9 medium on pigment production, cells were inoculated in production medium having one component missing as per Table 3. Treatments were kept on shaker set at 100 rpm, at 25 ± 2 °C in dark conditions.

**Table 3:** Effect of PGRs on callus induction

Treatment	Response	Treatment	Response	Treatment	Response	Treatment	Response
T1		T17		T33		T49	
T2	*	T18		T34		T50	
T3		T19		T35		T51	
T4		T20		T36		T52	
T5	*	T21		T37		T53	
T6	**	T22		T38		T54	
T7		T23	***	T39		T55	**
T8		T24		T40		T56	
T9	**	T25		T41		T57	
T10	****	T26		T42	**	T58	
T11		T27		T43		T59	
T12		T28		T44		T60	
T13		T29		T45		T61	
T14	****	T30		T46	***	T62	
T15	****	T31		T47		T63	
T16		T32	****	T48		T64	

NIL columns represent no swelling of explants and induction up to 40 days of incubation

* Swelling in all the explants after 20 days but induction was observed only in one explant after 30 days

** Swelling in all the explants after 15 days but induction was observed only in explants after 20 days

*** Swelling in all the explants after 20 days but induction was observed only in one explant after 30 days

**** Swelling in all the explants after 20 days but induction was observed only in one explant after 30 days

### Spectrophotometric analysis of shikonin derivatives

The quantification of shikonin derivatives was done at 2-day intervals in all the treatments. To release the pigment from cells, suspension cultures were sieved through 40 μm mesh and centrifuged for 15 min at 10,000*g* in Eppendorf tubes containing iso-amyl alcohol. The shikonin derivatives in the organic phase obtained being recovered as a blue aqueous solution by using 1 ml of 2.5 % KOH. Absorbance of samples was recorded at 620 nm using spectrophotometer (Hussain et al. 2012, 2011).

#### Standard shikonin solution

A total of 1 mg shikonin was dissolved in 2 ml iso-amyl alcohol to make 500 ppm solution. This solution was diluted 50 times to make 10 ppm solution (0.01 mg/ml). 200 μl of each concentration of shikonin solution was taken in 2 ml Eppendorf tube and 1 ml of KOH (2.5 %) was added to it. The solution was centrifuged at 10,000*g* for 15 min and allowed to stand for 10–15 min. The upper oily layer was removed and OD of blue colored solution thus obtained recorded at 620 nm. Blank was prepared with 200 μl iso-amyl alcohol instead of shikonin solution; all other steps being as stated above. This calibration curve was used to determine shikonin derivatives in cells and culture medium.

### Scale-up in bioreactor

#### Shake-flask experiments

Shake-flask experiments were performed with 10 % inoculum density in 250 ml glass Erlenmeyer flasks containing 50 ml of GM or PM for the pigment production. Two types of bioreactors were used as mentioned below:

#### Stirred-tank bioreactor

The modular bench-top, water-jacketed, glass vessel stirred-tank bioreactor (Bio Flo 110) having 2 L working volume was used for scale-up studies. The medium agitation was done at 100 rpm using top-driven motor having six-blade turbine impellers. The parameters like temperature (25 ± 2 °C) and pH (5.75 for GM and 6.00 for PM, prior to autoclaving) were set at the value as optimized during shake-flask experiments. The dissolved oxygen was supplied at 2 l min^−1^ using compressed air. Proportional integral derivative (PID) of biocontroller unit controlled the set parameters during bioreactor cultivation. The inoculation was done in sterile conditions under laminar airflow to avoid contamination.

#### Air-lift bioreactor

The bench-top, water-jacketed with internal concave bottom section glass vessel air-lift bioreactor (Biostat Bplus) of 2 L working volume was also used for scale-up studies. Temperature was set at 25 ± 2 °C, pH (set at 5.75 for growth medium and 6.00 for production medium before autoclaving) and DO is supplied with compressed air at 2 L min^−1^. The culture medium is mixed by gassing, where the air is introduced near the bottom via a sparger ring and rises through an internal draft tube (Table 4). Inbuilt PID controller with the bioreactor system controlled and monitored the set parameters during cultivation.

**Table 4:** Effect of initial pH of medium on shikonin production

Sr. no.	Time interval (days)	Shikonin (μg/ml)							
		pH 3.75		pH 5.75		pH 7.75		pH 9.75	
		Mean	SD	Mean	SD	Mean	SD	Mean	SD
1	0	0.5	0.096	0.525	0.082	0.525	0.035	0.5	0.072
2	2	0.75	0.075	1.25	0.121	1.05	0.095	1.275	0.197
3	4	1.025	0.109	2.25	0.252	1.825	0.175	2.6	0.319
4	6	2.375	0.218	5.025	0.229	3.05	0.353	7.05	0.321
5	8	3.2	0.229	5.8	0.409	4.675	0.303	9.3	1.029
6	10	5.9	0.377	7.75	0.804	7.5	0.750	9.85	0.721
7	12	6.15	0.242	8.375	0.533	8.8	0.393	10.025	0.801

### Statistical analyses

One-way ordinary ANOVA was performed for analyzing differences in shikonin production under (1) combinatorial salt treatment (2) combination of illumination and dark conditions. ANOVA was performed using GraphPad Prism (Version 5.0) statistical software at a significance level (*α*) of 0.05.

## Results and discussion

### Establishment of in vitro callus cultures and callus induction

**Fig. 1:**
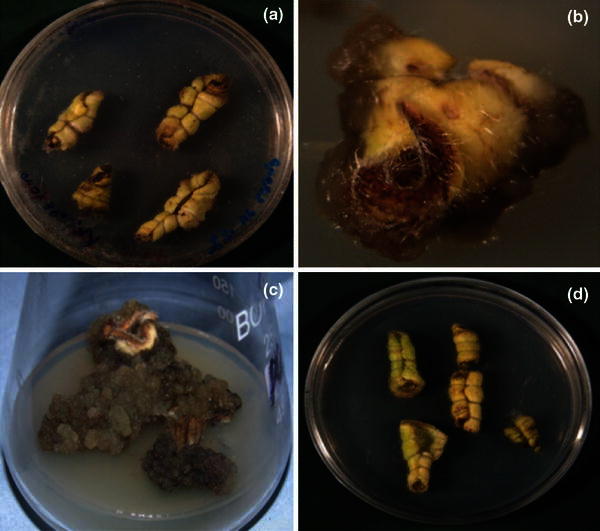
Leaf explants of *Arnebia* sp. for callusing **a** swelling of leaves, **b** initiation of callus (T14), **c** proliferation of callus (T15), and **d** control

The leaf segments were inoculated in Petri plates containing MS medium supplemented with different PGR combinations for callus induction (Fig. 1) and their effect on callus induction was observed at different time intervals (Table 3). Highest frequency of callus induction was recorded in T10, T15 and T32 after 35–40 days. However, lowering the auxin concentration in T14 resulted in early induction, i.e., 25–30 days. In these treatments, all leaf segments showed callus induction. About 50 % callus induction frequency was observed in MS medium supplemented with Kn (2 μM) and 2,4-D (2 μM), BAP (5 μM) and 2,4-D (2 μM) and DICAMBA (2 μM) and 2,4-D (2.5 μM). However, further proliferation was very slow in all the treatments tried and failed to produce friable callus. In basal MS medium (control) only swelling of leaf segments was observed, which later on turned brown.

### Growth kinetics

#### Settled cell volume (SCV)

**Fig. 2:**
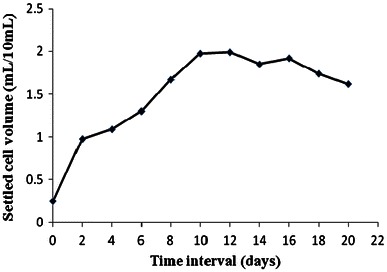
Growth curve based on settled cell volume (SCV)

The results showed that the cells take about 2 days (lag phase) after inoculation to start growing actively. The exponential growth (log phase) persist for about 8 days (2–10 days of cultivation period), as evident from the Fig. 2. Thereafter the growth starts ceasing to follow stationary phase (between 10 and 16 days of cultivation) and onset of decline phase (begins from 16th day onwards). Hence, the results showed that the *Arnebia* cells have 10 days of cultivation period before fresh sub-culturing for further multiplication can be done in liquid medium.

#### Fresh and dry weight

**Fig. 3:**
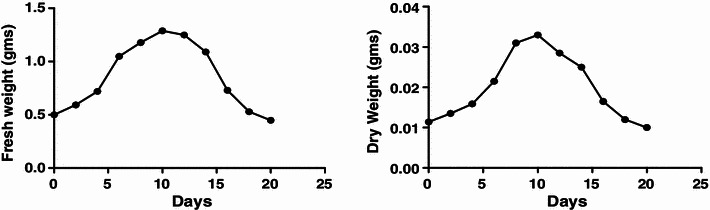
Growth curve based on fresh and dry cell weight

The growth kinetics based on fresh and dry weight was also studied. This is more accurate method than SCV, as in this actual increase in weight being recorded. The results showed a lag phase up to 1 day, log (exponential) phase from 2nd to 8th day, stationary phase from 8th to 12th day and decline phase 12th day onwards (Fig. 3). The reduced lag phase and early log phase can be corroborated to the age of inoculum used, making fast cell acclimatization.

### Pigment production

The already established two-stage culture system was used for pigment production. The cells grown in GM were transferred to modified M9 medium at 10 % inoculum density, as described in “Materials and methods”. The cultures were kept under dark conditions at 25 ± 2 °C on shaker.

### Factors affecting cell growth and pigment production in suspension culture

#### Effect of illumination

##### Cell growth

**Fig. 4:**
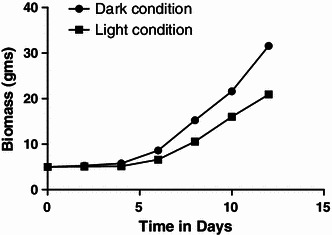
Effect of dark and light conditions on biomass production

**Fig. 5:**
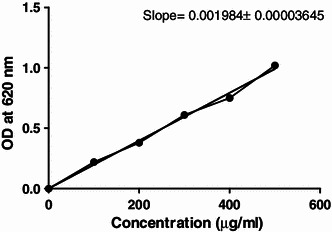
Standard curve of shikonin

The cells grown in GM under 24 h light as well as dark conditions revealed that cell biomass grows almost six-times higher under dark condition and increased from 5 to 31.58 g as compared to 20.91 g in the presence of light, at the end of cultivation period (Figs. 4, 5). The per cent increase in yield was calculated to be 531 %.

##### Pigment production

**Fig. 6:**
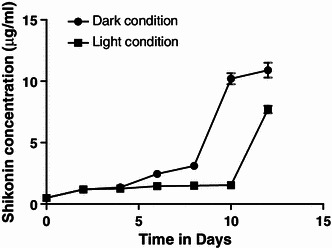
Comparison of dark and light conditions on shikonin production

The cells were inoculated in modified M9 medium under 24 h light as well as dark conditions to observe effect of illumination on pigment production. There was an increase in pigment content irrespective of light conditions till 4th day of culture but as the culture period progresses, the pigment content decreases in the presence of light, whereas in dark it continued to increase. Dark red colored pigment was observed at the end of culture period in suspension culture growing under dark conditions. However, there was very less pigment formation in cells growing under the presence of light. This shows that light completely inhibits the production of pigment during earlier phase of cultivation period (Fig. 6). Results revealed that maximum shikonin content (OD_620_ = 0.218) was observed in dark conditions after 12 days of culture period as compared to light (OD_620_ = 0.120) (Fig. 6).

##### Effect of illumination exposure period on pigment yield

**Fig. 7:**
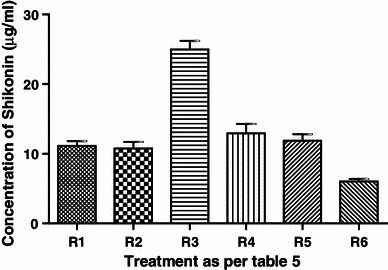
Effect of light exposure period on shikonin production

Results showed that maximum pigment production was obtained when the cell suspension culture was kept in light for 6 days and then in dark for 4 days (Fig. 7) than rest of the illumination dark period combination.

The decrease in pigment production in cultures grown under the presence of light is attributed to the inhibition of *p*-hydroxybenzoic acid geranyl transferase (PGT) activity—a key enzyme of shikonin biosynthesis Irradiation results in accumulation of *o*-glucosidic form of *p*-hydroxybenzoic acid (PHB). PHB is stored in vacuole of cells in its glucoside form in presence of light and upon transfer of cells to dark condition, this glucoside is hydrolyzed to give free hydroxybenzoic acid, which on further reaction metabolized to shikonin biosynthesis (Sommer et al. 1999; Xuqing and Dewei 1998).

The maximum production of naphthoquinone pigment was observed within 10 days of cultivation period, thereafter it declined. The decline was possibly due to cell death or as result of feedback inhibition caused by the accumulation of pigment. Dark condition was a pre-requisite for the biosynthesis of pigment as there was no pigment production in light. This is due to the fact that these compounds are accumulated in the underground part of the plant.

#### Effect of pH

The pH of the medium was found to have pronounced effect on cell biomass and pigment production. Cell growth is directly influenced by the nutritional composition of the medium, but the uptake of nutrient is mainly influenced by pH of the medium. The cell growth is directly influenced by the nutritional composition of the medium, but the uptake of nutrient is mainly influenced by pH of the medium. The various biochemical reactions occurring at the cellular level are also influenced by change in pH due to the specificity of pH-dependent enzymes.

##### Cell growth

**Fig. 8:**
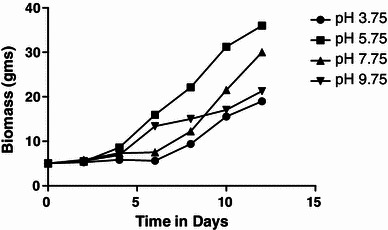
Effect of initial pH of medium on biomass growth

The *Arnebia* cells were cultured in GM under different pH regime to observe its effect on biomass growth. Results revealed that cell biomass recorded almost seven-times higher growth at pH 5.75 and increased from 5 to 35.99 g as compared to 18.97 g at pH 3.75, after 12 days of cultivation period. The increase in yield was found to be 620 %. As the pH of the medium increased from 5.75 to 9.75, the cell growth was found to decrease. At pH 5.75, an increase in fresh weight of cell biomass was observed throughout the cultivation period. However, a low biomass yield was recorded in extremes of pH, i.e., 3.75 and 9.75 (Fig. 8). The pattern of cell growth is indicative of 10 days sub-culturing period for maximum biomass production in cell suspension cultures.

##### Pigment production

**Fig. 9:**
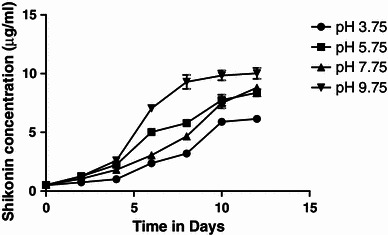
Effect of initial pH of medium on shikonin production

The cells were grown in M9 medium under different pH regime. Results revealed that the cells growing at high pH (9.75) exhibited high and early synthesis of pigment (6 days). The maximum shikonin content (OD_620_ = 0.210) was observed at pH 9.75, while the lowest value (OD_620_ = 0.123) was observed at pH 3.75 (Fig. 9). Cell growth and production of shikonin was observed to be greatly influenced by the pH of the respective media (Table 5). Acidic pH favors cell growth, whereas alkaline pH favors pigment production. It was observed that pH of the growth medium as well as that of production medium changed by 1–2 Units after autoclaving. The change was more pronounced in production medium because of the low buffering capacity of the medium. The change in pH after autoclaving is due to the high during sterilization temperature, which alters the composition of various nutrients and salts of the medium. The initial increase in pH value might result from the fast uptake and reduction of nitrates (formation of OH^−^) while acidification of the medium at later stages of cell culture can be associated with the uptake of ammonium ion. Increased production of pigment at alkaline pH of production medium could be correlated with the enhanced activity of PGT, one of the key regulatory enzymes of shikonin biosynthetic pathway. This enzyme catalyzes the condensation of *p*-hydroxybenzoic acid and geranyl pyrophosphate (GPP) to yield *m*-gernayl-*p*-hydroxybenzoic acid (GBA) requires the optimum pH which ranges from 7.1 to 9.3 for its activity (Malik et al. 2011; Xuqing and Dewei 1998).

**Table 5:** Production of shikonin under dark and light conditions

Sr. no.	Time interval (days)	Shikonin (μg/ml)			
		Dark condition		Light condition	
		Mean	SD	Mean	SD
1	0	0.5	0.132	0.5	0.055
3	2	1.2	0.264	1.2	0.132
3	4	1.35	0.229	1.25	0.079
4	6	2.45	0.132	1.45	0.11
5	8	3.1	0.360	1.49	0.127
6	10	10.2	0.781	1.55	0.125
7	12	10.9	1.044	7.7	0.529

#### Effect of M9 medium constituents on pigment production

**Fig. 10:**
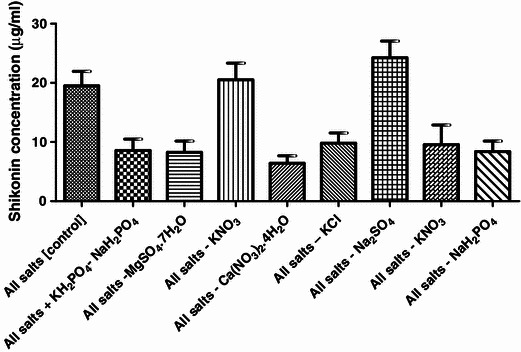
Effect of salts on production of shikonin

The purpose of this experiment was to study the effect of individual constituents of M9 medium (Table 3) on pigment production. Na_2_SO_4_ is used at higher concentration (1,480 mg l^−1^) in M9 medium. It was interesting to notice that its exclusion of Na_2_SO_4_ (T7) does not affect the pigment yield and recorded a little higher OD value (OD_620_ = 0.141) as compared to T1, i.e., control (OD_620_ = 0.112). However, the exclusion of other constituents does not reveal any pronounced effect on metabolite yield. TSS and pH were found to be higher in combination where MgSO_4_·7H_2_O (T3) was missing (Fig. 10).

### Scale-up in bioreactor

**Fig. 11:**
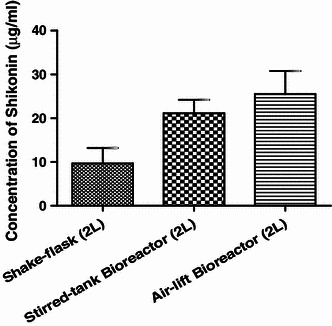
Comparative histogram for produce of shikonin in 3 different culture systems

Experiments on scale-up of cell biomass production (GM) and pigment production (modified M9) from shake flask to bioreactor was done with optimized parameters as discussed in “Materials and methods” section. The data were recorded for cell biomass and pigment production in shake-flask and different bioreactors. Results showed that the cell biomass was drastically decreased with the scale-up from shake-flask (1,249.2 g) to stirred-tank bioreactor (480 g) and air-lift bioreactor (450 g). However, the pigment production as evident from Table 6 is considerably higher in air-lift reactor as well as stirred-tank bioreactor (Fig. 11).

**Table 6:** Production of shikonin in three different culture systems

Sr. no.	Culture system	Concentration of shikonin produced (μg/ml)	
		Mean	SD
1	Shake-flask (2 L)	9.7	3.522
2	Stirred-tank bioreactor (2 L)	21.15	3.050
3	Air-lift bioreactor (2 L)	25.5	5.286

### Statistical analyses

ANOVA table for evaluating the effect of M9 media constituents (as per Table 7) is given in Table 8. From Table 8 it is revealed that there is a significant effect (*P* < 0.001) of M9 media constituents on pigment production.

**Table 7:** Combination of salts used for studying effect on pigment production

Sr. no.	No. of combinations	Shikonin concentration (μg/ml)	
		Mean	SD
T1	All salts [control]	19.5	2.438
T2	All salts + KH_2_PO_4_–NaH_2_PO_4_	8.55	1.931
T3	All salts: MgSO_4_·7H_2_O	8.25	1.920
T4	All salts: KNO_3_	20.5	2.825
T5	All salts: Ca(NO_3_)_2_·4H_2_O	6.4	1.264
T6	All salts: KCl	9.8	1.739
T7	All salts: Na_2_SO_4_	24.25	2.818
T8	All salts: KNO_3_	9.55	3.308
T9	All salts: NaH_2_PO_4_	8.4	1.757

**Table 8:** One-way ANOVA of effect of salts combination as per Table 7

*P* value	*P* < 0.0001		
*P* value summary	***		
Are means significant different? (*P* < 0.05)	Yes		
Number of groups	9		
*F*	24.91		
*R* squared	0.9172		
ANOVA table	SS		
Treatment (between columns)	1,062	df	MS
Residual (within columns)	95.9	8	132.7
Total	1,158	18	5.328
		26	

Table 9 gives the ANOVA statistics for data collected after exposing the cell culture to light conditions for different duration. A very significant correlation (*P* < 0.001) is obtained between light exposure duration and pigment yield and ANOVA revealed that there is a very less probability that the variance among the means of obtained result is due to chance.

**Table 9:** One-way ANOVA of effect of light exposure duration as per Table 2

*P* value	*P* < 0.0001		
*P* value summary	***		
Are means significant different? (*P* < 0.05)	Yes		
Number of groups	6		
*F*	42.04		
*R* squared	0.946		
ANOVA table	SS	df	MS
Treatment (between columns)	605	5	121
Residual (within columns)	34.54	12	2.878
Total	639.5	17	

## Conclusion

The present study was carried out to exploit the potential of *Arnebia* sp. for naphthoquinone pigment production through cell culture. Growth kinetics was studied by settled cell volume and fresh/dry cell weight methods. It was found that fresh/dry weight was more efficient to predict the growth phases.

Effect of factors like pH, illumination and medium constituents affecting the cell growth and pigment production was also studied. Different factors affecting the cell growth and pigment production were studied. It was concluded that dark conditions at room temperature (25 ± 2 °C) and acidic pH (5.75) favors the cell growth in GM while alkaline pH at same conditions favors the pigment production. In case of M9 medium constituents, interestingly, Na_2_SO_4_ does not affect the pigment yield and was found at par with control. During scale-up, the cell biomass productivity decreases drastically; however, the pigment yield increased.

Finally, statistical analyses of the obtained data through ANOVA rule out any chance of random differences among the final production of pigment production and establish the fact that there is effect of constituents of M9 medium and illumination on the pigment yield.
